# Optogenetic control of human neurons in organotypic brain cultures

**DOI:** 10.1038/srep24818

**Published:** 2016-04-21

**Authors:** My Andersson, Natalia Avaliani, Andreas Svensson, Jenny Wickham, Lars H. Pinborg, Bo Jespersen, Søren H. Christiansen, Johan Bengzon, David P.D. Woldbye, Merab Kokaia

**Affiliations:** 1 Epilepsy Centre, Department of Clinical Sciences, Lund University Hospital, Lund, Sweden; 2 Lund Stem Cell Center, BMC B10 and Department of Neurosurgery, Lund University Hospital, Lund, Sweden; 3 Epilepsy Clinic & Neurobiology Research Unit, Rigshospitalet, Copenhagen University Hospital, University of Copenhagen, Denmark; 4 Department of Neurosurgery, Rigshospitalet, Copenhagen University Hospital, Denmark; 5 Laboratory of Neural Plasticity, Department of Neuroscience and Pharmacology, University of Copenhagen, Denmark

## Abstract

Optogenetics is one of the most powerful tools in neuroscience, allowing for selective control of specific neuronal populations in the brain of experimental animals, including mammals. We report, for the first time, the application of optogenetic tools to human brain tissue providing a proof-of-concept for the use of optogenetics in neuromodulation of human cortical and hippocampal neurons as a possible tool to explore network mechanisms and develop future therapeutic strategies.

Optogenetic tools are used in an increasing number of studies and have successfully resolved many unknown details related to the role of different populations of neurons in mechanisms governing normal brain function, such as learning and memory^1^,^2^, circadian rhythms^3^,^4^,^5^,^6^ and visuospatial discrimination^7^. Moreover, successful attempts have been made at understanding the pathophysiology of neural diseases in animal models, and possibilities for prosthetic correction of impaired brain function by diverse optogenetic approaches^8^,^9^,^10^. However, until now, it still remained unclear whether an optogenetic approach would be feasible in human brain tissue as an important step towards developing alternative treatment strategies for severe neurological diseases. Here we demonstrate that neurons in brain tissue derived from adult human temporal lobe neocortex or hippocampus resected because of medically intractable epilepsy, are capable of expressing ChR2, one of the main excitatory opsins widely used for optogenetic studies in animals. Furthermore, we show that human neurons expressing ChR2 are activated by blue light to generate action potentials.

Temporal neocortical (NC) tissue and human hippocampus (HPC) were obtained by surgical resections (4 NC/4 HPC) from seven patients treated for intractable epilepsy at the Department of Neurosurgery of Lund University Hospital, Sweden, and Rigshospitalet in Copenhagen, Denmark. The procedures and use of resected human brain tissue were approved by the local Ethical Committee in Lund, (#212/2007) and Copenhagen (H-2-2011-104) in accordance with the Declaration of Helsinki and written informed consent were obtained from all subjects prior to surgery. Neocortex or hippocampus were surgically removed *en bloc* and immediately submerged in carbonated ice-cold sucrose cutting solution and transferred from the operating room to the laboratory, where 250 μm thick slices of temporal lobe neocortex and the hippocampal formation were prepared and cultured under standard slice culturing conditions^11^. The slices were allowed to settle for 12 h, and thereafter lenti-viral vector containing ChR2 gene under the human synapsin promoter (LV-Syn-hChR2(H134R)-eYFP) was added. See supplementary information for extended materials and methods.

After two weeks of culturing, slices were transferred to the recording chamber and ChR2 transduction of neurons in the human organotypic brain cultures was identified by expression of eYFP, which was attached as a label to the ChR2 viral vector construct (Figs 1a–c and 2a,b). The ChR2 expression was widespread, covering most of the cultured slice areas, and was localized in cells with neuronal appearance, i.e. clearly identifiable soma and dendrites and co-stained for microtubule-associated protein, MAP2 (Fig. 1a–c). Quantification of the percentage of cells co-expressing MAP2 and eYFP in 23 cultured slices revealed that a large portion of these cells were neuronal (60.4 ± 3.9% of eYFP positive cells).

Whole-cell patch-clamp recordings were performed from eYFP-expressing neurons (Fig. 2a,b) to test for functional properties and functional state. The majority of the recorded neurons (45 of 63) showed a resting membrane potential more negative than −45 mV, measured immediately after breaking into the whole-cell mode. This is in line with our previous recordings from neurons in acute temporal cortical slices^12^. Neocortical and hippocampal neurons did not differ from each other with regard to the magnitude of resting membrane potential (−60.2 ± 2.7 mV, n = 24 and −61.5 ± 2.2 mV, n = 22, respectively, P = 0.71) nor in their input resistance (207 ± 43 MΩ, n = 24 and 232 ± 29 MΩ, n = 23, respectively, P = 0.46). Twenty-one neurons of 45 generated action potentials when step-depolarized by current injection, with average amplitude of 54.0 ± 17.3 mV and duration of 3.1 ± 0.3 ms for neocortical cells (n = 13, Fig. 2c) and 34.0 ± 6.2 mV and duration of 3.6 ± 0.5 ms for hippocampal cells (n = 8, Fig. 2d). These characteristics were closely comparable to those of human neurons recorded in acute neocortical^12^ and hippocampal slices^13^. **E**lectrical stimulation of CA1 stratum radiatum fibers induced EPSCs (often poly-synaptic), confirming functional synaptic afferents to these neurons. Taken together, these data show that the majority of the neurons in organotypic cultures were relatively healthy and exhibited normal neurophysiological intrinsic properties.

Since neurons from hippocampal and cortical slice cultures did not differ with regard to their intrinsic properties (P = 0.28 for AP-amplitude and 0.38 for AP-duration), the data on optogenetics from these neurons were pooled together. When slices were exposed to 470 nm-blue LED-light delivered though the 40X-objective, ChR2 expressing neurons responded by depolarization with a typical initial peak current followed by a lower steady-state depolarization (783.9 ± 100.0 pA and 536.5 ± 69.4 pA, respectively, n = 31; see Fig. 2e,g). Moreover, in current-clamp mode, action potentials induced by light stimulation were also frequently observed (Fig. 2f). Repetitive light-pulse stimulations reliably induced depolarizing currents, (827.6 ± 150.6 pA, n = 16, Fig. 3a). Paired-pulse light stimulation with 100 ms interval (with a peak amplitude first response of 952.6 ± 134.7 pA, n = 32) revealed a decrease in amplitude of the second pulse-induced response with the ratio to the first of 0.7 ± 0.1 (n = 32, see Fig. 3b). These currents were mainly generated by ChR2 activation and partly by synaptic inputs from other light-activated neurons, since addition to the perfusion medium of AMPA receptor blocker NBQX and NMDA receptor antagonist AP5 alone, or in combination with GABA_A_-receptor blocker PTX, resulted in a small reduction of the light-induced currents (to 87.3 ± 4.9%, n = 5, p = 0.04 and 71.5 ± 4.1% of control, n = 3, p = 0.03, respectively; see Fig. 3c) with no change of the paired-pulse ratio in the presence of antagonists (normalised to baseline, in AMPA and AP5, 99.4 ± 0.4%, n = 5, P = 0.26 and in AMPA, AP5 and PTX, 99.6 ± 0.9%, n = 3, P = 0.71), suggesting that reduction of the second light pulse-induced response was mainly due to ChR2 activation/inactivation dynamics^14^. Overall, these data show that (i) neurons in cultured human epileptic tissue slices can survive for prolonged periods, up to 14 days with relatively normal physiological function, (ii) that these neurons can effectively express ChR2 and respond to light stimulation by generation of action potentials, and (iii) these light-generated action potentials can induce synaptic responses in the neighbouring neurons.

Human brain tissue has been shown to survive for several hours after resection as acute temporal lobe or cortical slices^15^,^16^, allowing for electrophysiological investigations of neuronal function. Post-mortem brain tissue in culture has been reported to retain morphological characteristics up to four weeks after culturing and express β-galactosidase delivered by an AAV-vector^17^. Electrical stimulation can induce epileptic activity under slice culture conditions for up to 30 days^18^. However, our study is the first demonstration, to our knowledge, of whole-cell electrophysiological recordings from human epileptic tissue successfully cultured in standard medium for up to 2 weeks. In addition to previous studies, this finding is a significant contribution for establishing long-term survival of human brain slices, which would enable certain experimental approaches, e.g. transgene expression studies. This approach also opens up new possibilities for exploring relatively long-term treatment outcomes, such as for antiepileptic drugs or various other treatment strategies. Most importantly, however, our data demonstrate that viral vector-based optogenetic tools can be used to express opsins, such as ChR2, in human neurons to the level that enables functional manipulation of these neurons. Optogenetics has previously been applied to nonhuman primates^19^,^20^. In these studies, focus was mainly set on clarifying mechanisms within the visual system^7^,^21^. Collectively, previous primate studies demonstrate that the optogenetic approach reveals intimate mechanisms of network activity associated with certain behaviour of the neuronal systems, which may be specific to primates or humans. Therefore, our study provides an important proof-of-concept that optogenetics can become a feasible approach to explore human brain tissue *ex vivo*, where networks are at least partially preserved, as well as to develop future alternative therapeutic strategies for neurological diseases, such as e.g. epilepsy.

## Additional Information

**How to cite this article**: Andersson, M. *et al.* Optogenetic control of human neurons in organotypic brain cultures. *Sci. Rep.* **6**, 24818; doi: 10.1038/srep24818 (2016).

## Supplementary Material

Supplementary Information

## Figures and Tables

**Figure 1:**
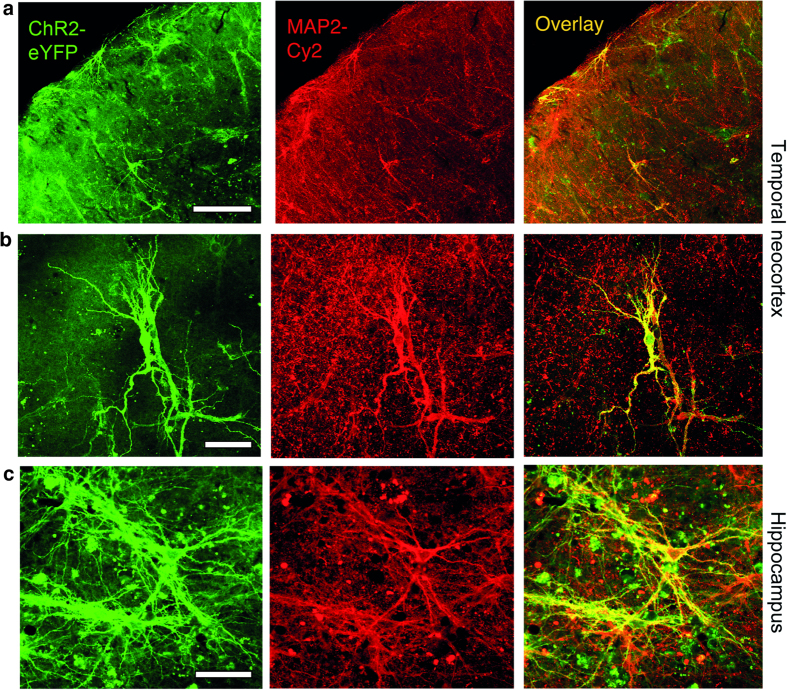
ChR2 expression in human organotypic brain slice cultures. Representative confocal images of enhanced yellow fluorescent protein expression (eYFP, green), neuron-specific microtubule associated protein-2 (MAP2, red) and the overlay of both channels. (**a**) Left, middle and right image from a cortical organotypic tissue culture (scalebar 200 μm) with a higher magnification image in (**b**) (scalebar 50 μm). (**c**) Left, middle and right image from a hippocampal organotypic tissue culture (scalebar 50 μm).

**Figure 2:**
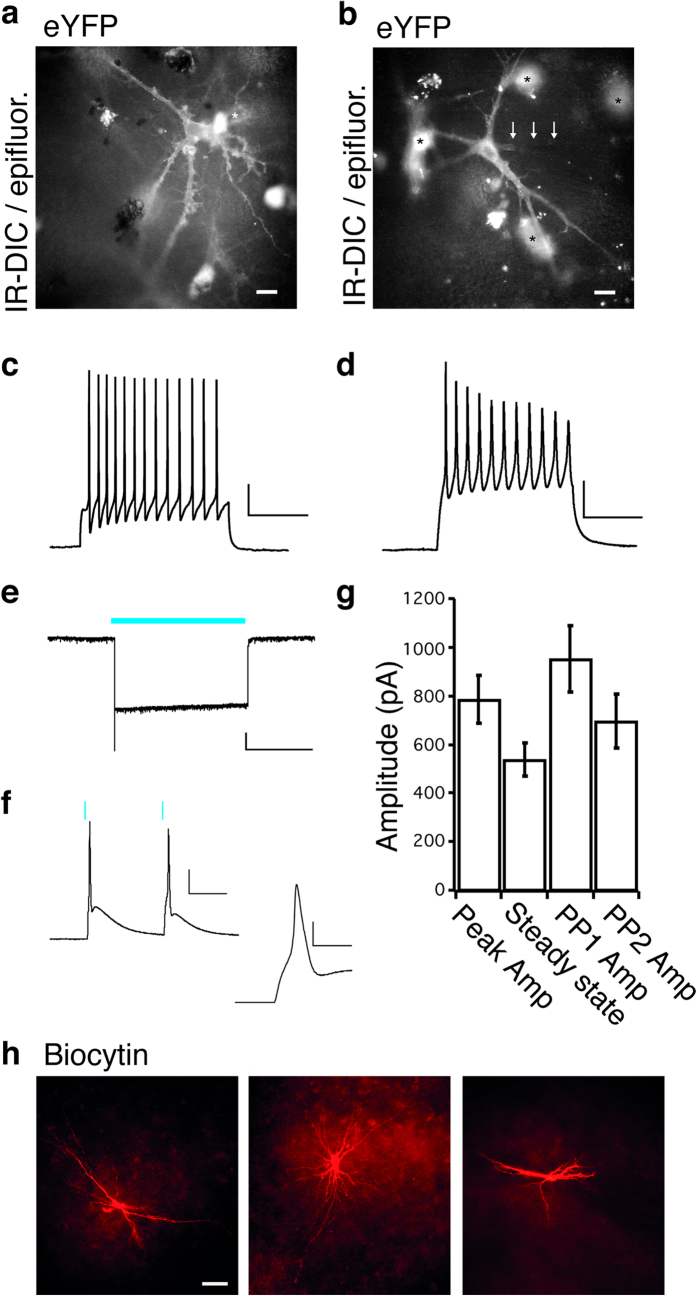
Light-induced responses of ChR2-expressing neurons in human organotypic brain tissue cultures. ChR2-expressing cells were identified for whole-cell patch-clamp recordings with the expression of the reporter EYFP in infrared-differential interference contrast (IR-DIC) microscopy combined with epifluorescence exemplified in (**a**) a cultured temporal neocortical neuron and in (**b**) a cultured hippocampal neuron (scale bars 20 μm). Neurons in both preparations, temporal neocortical neuron (**c**) and hippocampal neuron (**d**) fired action potentials in response to a depolarising current step (scale bars 20 mV, 200 ms). (**e**) A 10-s continuous blue light-pulse induced an inward current in voltage clamp (scale bar 50 pA, 5 s) and in (**f**) paired 1 ms light pulses elicited action potentials in current-clamp (scale bar 20 mV, 50 ms and 5 ms in inset). (**g**) Average current obtained from hippocampal and temporal neocortical neurons induced by blue light application, measured as peak amplitude (peak amp) or steady state (2 s after peak) evoked by a 5 s light pulse (n = 31) or peak amplitude in first response (PP1) or second response (PP2) after paired 1 ms light pulses, 100 ms interval (n = 32). (**h**) Biocytin staining showing three representative patterns of dendritic morphology from whole-cell recorded neurons (scale bar 50 μm).

**Figure 3:**
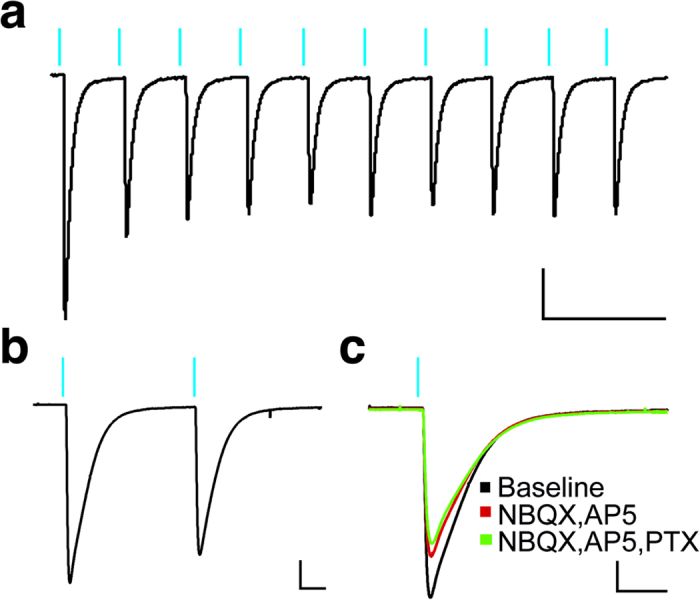
Light-induced currents in ChR2-expressing neurons in the presence of glutamate and GABA receptor antagonists. (**a**) A 10-pulse train (1 ms at 50 Hz) of blue light consistently elicited inward currents in voltage-clamp mode. (**b**) Paired-pulse light stimulation with a 100 ms interval (scale bar 200 pA, 20 ms). (**c**) Representative traces showing that a large part of the light-induced currents remained after AMPA (NBQX), NMDA-(AP5) and GABA_A_-(PTX)-receptor blockade (scale bar 200 pA, 20 ms).
