# Supplementary material for: Optogenetic control of human neurons in organotypic brain cultures
Source: Sci Rep. 2016 Apr 21;6:24818. doi: 10.1038/srep24818 (PMC4838935; doi:10.1038/srep24818)
Supplement: Supplementary Information [file srep24818-s1.pdf]

Supplementary information

Optogenetic control of human neurons in organotypic brain cultures

My Andersson[1*], Natalia Avaliani[1], Andreas Svensson[4], Jenny Wickham[1], Lars H. Pinborg[3], Bo Jespersen[5], Søren H. Christiansen[2], Johan Bengzon[4], David P.D. Woldbye[2], Merab Kokaia[1]

[1] Epilepsy Centre, Department of Clinical Sciences, Lund University Hospital, Lund, Sweden

[2] Laboratory of Neural Plasticity, Department of Neuroscience and Pharmacology, University of Copenhagen, Denmark

[3] Epilepsy Clinic & Neurobiology Research Unit, Rigshospitalet, Copenhagen University Hospital, University of Copenhagen, Denmark

[4]Lund Stem Cell Center, BMC B10 and Department of Neurosurgery, Lund University Hospital, Lund, Sweden

[5] Department of Neurosurgery, Rigshospitalet, Copenhagen University Hospital, Denmark

***Corresponding author** My Andersson, **correspondence to my.andersson@med.lu.se.**

Extended material and methods

Ethics

The procedures and use of resected human brain tissue was approved by the local Ethical Committee in Lund, (#212/2007) and Copenhagen (H-2-2011-104), in accordance with the Declaration of Helsinki and informed consent were obtained from all patients recruited to the study.

Human organotypic slice preparation

The human temporal lobe (hippocampus - HPC) and neocortical (NC) tissues were obtained by surgical resections (4 NC/ 4 HPC) from seven patients treated for intractable epilepsy at the Departments of Neurosurgery of Lund University Hospital, Sweden, and Rigshospitalet in Copenhagen, Denmark. The resected tissue was immediately submerged in ice-cold sucrose solution containing in mM: 200 sucrose, 21 $NaHCO_3$, 10 glucose, 3 KCl, 1.25 NaH_2PO_4, 1.6 $CaCl_2$, 2 $MgCl_2$, 2 $MgSO_4$ (all from Sigma-Aldrich, Sweden), bubbled with carbogen (O_2, 95% and CO_2, 5%) adjusted to 300-310 mOsm and 7.4 pH and transferred from the surgical theatre to the laboratory. Time of transport of tissue from Copenhagen Hospital operating theatre to Lund laboratory was approximately one hour. In our pilot experiments and in those published previously, to ascertain that the resected brain tissue was not deteriorating during the transportation from Copenhagen, we compared acute whole-cell and field recordings of slices prepared from Copenhagen resection to those obtained from Lund Hospital, and could not find any obvious difference between these (see Ledri et al, 2015).

250 μm thick coronal slices were cut on a Leica VT1200 vibratome in the same sucrose-solution as mentioned above. The slices were transferred to ice-cold rinsing medium containing Hank's balanced saline solution (HBSS) with HEPES, 20mM, glucose, 17.5 mM and 0.5% penicillin/streptomycin solution (all from Life Technologies, Thermo Fisher Scientific Inc, Sweden) before placing them on cell culture inserts (Millipor, Sweden, number PICM03050) in 6-well plates

with 960 µl equilibrated culturing medium. This medium contained 50% minimum essential media (MEM), 25% horse serum, 18% HBSS, and 2% B27 supplemented with 0.5% penicillin/streptomycin solution (all from Life Technologies, Thermo Fisher Scientific Inc, Sweden), glutamine 2 mM, glucose 11.8 mM, and sucrose 20 mM (all from Sigma-Aldrich, Sweden). Slices were cultured as interface cultures at 37°C, 5% CO_2, and ambient O_2 in 90% humidity. Medium was changed on day 1 of culturing and three times per week thereafter. B27 was withdrawn from the medium after 1 week of culturing.

Channelrhodopsin expression

The slices were allowed to settle for 12 h, and thereafter lenti-viral vector containing ChR2 gene under the human synapsin promoter (LV-Syn-hChR2(H134R)-EYFP; Addgene plasmid #20945) was applied as a drop directly on the top of the slices (titer of 3.1x10^7 genomic particles/ml).

Electrophysiological recordings

After two weeks of culturing, slices were excised on their culturing membranes and transferred to the recording chamber for whole-cell patch-clamp recordings. Individual slices were placed in a submerged recording chamber and infrared differential interference contrast microscopy (IR-DIC, Hamamatsu, Japan) was used together with a 460 nm LED-light (Prizmatix, USA) for visual identification of ChR2-expressing neurons. The slices were continuously perfused at a rate of 3 ml / minute with artificial cerebrospinal fluid containing in mM: 129 NaCl, 21 $NaHCO_3$, 10 glucose, 3 KCl, 1.25 NaH_2PO_4, 2 $MgSO_4$, 1.6 $CaCl_2$, adjusted to 300-310 mOsm and 7.4 pH, and constantly oxygenated and maintained at 32-34 °C. For whole-cell patch-clamp recordings, glass pipettes were back-filled with a solution containing (in mM): K-gluconate 122.5, KCl 12.5, KOH-HEPES 10, KOH-EGTA 0.2, MgATP 2, Na_3GTP 0.3, NaCl 8, (pH 7.2-7.4, mOsm 290-300) and had a tip resistance of 3-5 MΩ. Pipette current was corrected online before gigaseal formation while fast capacitive currents were compensated for during cell-attached configuration. Biocytin (5 mg/ml)

was always included in the pipette solution for post-hoc identification of the recorded cells.

Resting membrane potential (RMP) was recorded in current clamp mode at 0 pA immediately after establishing whole-cell configuration. Series resistance and input resistance (Ri) were calculated from a 5 mV pulse. Series resistance was constantly monitored (average 16.4 ± 9.0 MΩ, n = 46) and if changed more than 20% during drug-application, the recordings were excluded from further analysis. Action potential (AP) threshold was determined by 500 milliseconds square current step injections at RMP, with 50 pA increments from 50 to 300 pA. Ramp injection of 1 s current was used to determine action potential threshold in addition to step depolarization. AP amplitude was measured from threshold to peak and duration was measured as the width at the threshold. Whole-cell currents were measured in voltage-clamp mode at a holding potential of -70 mV whereas membrane potential changes were measured in current clamp mode at RMP. 470 nm light-pulses were generated by a LED (Prizmatix, USA) and delivered via a 40X water immersion objective (Olympus, Germany) with a maximum energy density of 8.5 mW/cm2, in a 5 s long pulse, paired-pulses or in 10 repetitive pulses (both 1 ms, 100 ms apart) at a frequency of 0.067 Hz. NBQX (50 µM, Sigma Aldrich, Sweden) and AP5 (Abcam Biochemicals, UK) were applied to slices for blocking AMPA/kainate receptors and NMDA receptors, respectively, followed by the $GABA_A$-blocker picrotoxin (50 µM, Sigma Aldrich, Sweden). All data were acquired at a sampling rate of 10 kHz using Patchmaster Software and HEKA amplifies (EPC10 or EPC9, HEKA Elektronik, Lambrecht, Germany).

Immunohistochemistry and Imaging

After whole-cell patch-clamp recordings, the slices were fixed in 4% paraformaldehyde in phosphate buffer (PB) for 12-24 hours, rinsed in PB and then stored in anti-freeze solution (ethylenglycol and glycerol in PB) at -20°C. For immunohistochemistry, slices were washed three times in Kalium-PBS (KPBS), and incubated in 1%Triton X-100-KPBS (tKPBS)/10% normal goat

serum (NGS) for one hour followed by 1% tKPBS/10% NGS with primary antibody (Rabbit-anti GFP, 1:10 000, Abcam, UK, mouse-anti MAP2, 1.500, Sigma Aldrich, Sweden) for 48 hours in 4°C. Following, slices were rinsed three times in KPBS solution and incubated for 24 hours in 4°C in secondary antibody (Cy2-anti-Rabbit, 1:400 and Cy3-anti-mouse, 1:400) and Cy5-conjugated Streptavidin (Invitrogen, 1:300). They were subsequently washed and mounted on coated glass slides and cover slipped with DABCO. Images were analysed with a laser scanning confocal microscope (Leica).

Analysis and statistics

Response to light activation was analysed offline using Fitmaster (HEKA Elektronik, Germany) and Igor Pro (Wavemetrics, Lake Oswego, OR, USA) softwares. All data are expressed as means ± SEM and analyzed using Student's paired *t*-tests. The level of statistical significance was set at $p<0.05$.
